# Interactions of Morphine and Peptide 234 on Mean Plasma Testosterone Concentration

**DOI:** 10.5812/ijem.12554

**Published:** 2014-01-05

**Authors:** Fariba Mahmoudi, Homayoun Khazali, Mahyar Janahmadi

**Affiliations:** 1 Department of Animal Sciences,Faculty of Biological Sciences, Shahid Beheshti University, Tehran, IR Iran; 2 Department of Phsiology Neurophysiology Research Center, Medical School, Shahid Beheshti University of Medical Sciences, Tehran, IR Iran

**Keywords:** Kisspeptin, P234, Morphine, Testosterone

## Abstract

**Background:**

Kisspeptin-GPR54 system stimulates the hypothalamus-pituitary-gonadal (HPG) axis; dysfunction of the gene encoding the GPR54 receptor causes hypogonadism and infertility. Opioid peptides inhibit the reproductive axis. Peptide 234 is a GPR54 receptor antagonist and blocks the stimulatory effects of kisspeptin on HPG axis.

**Objectives:**

Interactions of morphine, kisspeptin and peptide 234 on mean plasma testosterone concentration was investigated in rats. .

**Materials and Methods:**

In the present experimental study, seventy male Wistar rats in 14 groups (n = 5 in each group) received saline, different doses of kisspeptin (100 pmol, 1 or 3 nmol, Intracerebroventricular (ICV)), P234 (1 or 2.5 nmol) or Co- administration of kisspeptin, P234, morphine and naloxone at 09:00 - 09:30 am. In the co-administrated groups, kisspeptin was injected at 15 min following P234, morphine or naloxone injections. Blood samples were collected 60 min following injections. Plasma testosterone concentration was measured using the rat testosterone ELISA kit.

**Results:**

Injections of kisspeptin (1 or 3 nmol) significantly increased the mean testosterone concentration compared to saline. Injection of different doses of P234 (1 or 2.5nmol) did not significantly decrease mean testosterone compared to saline. Co-administration of kisspeptin and different doses of P234 significantly decreased mean testosterone concentration compared to the kisspeptin group. Co-administration of P234/morphine or P234/naloxone significantly decreased mean testosterone concentration compared to kisspeptin/saline, kisspeptin/morphine or kisspeptin/ naloxone groups.

**Conclusions:**

Morphine and kisspeptin/GPR54 signaling pathway may interact with each other to control the hypothalamic-pituitary-gonadal axis.

## 1. Background

Narcotics and opioid peptides are known to inhibit the activity of the hypothalamic-pituitary-gonadal (HPG) axis in rodents, ruminants and humans. Central or peripheral injection of morphine or β- endorphin or other opiates significantly decreases mean plasma luteinizing hormone (LH) and circulating gonadal steroids including testosterone and estrogens, mainly via binding to opioid μ-type receptors (1-3). Naloxone, an opioid receptor antagonist competitively inhibits the effects of opioids on the HPG axis activity and increases LH and gonadal hormone secretions in both male and female members of different species (4-11). 

Kisspeptin neuropeptide is the product of Kiss1 gene. Like other peptide neurotransmitters/neuromodulators, kisspeptin is initially translated to a long kisspeptin precursor (145 amino acid), which is cleaved to form shorter mature peptides including kisspeptin 54 in human (kisspeptin 52 in rodents). The kisspeptin 54 or 52 amino acids are proteolytically cleaved to give rise to shorter products namely kisspeptin-14, kisspeptin-13 and kisspeptin-10 (12). Based on the prediction of a cleavage site and subsequent binding assay studies, it has been shown that the 10 amino acid “core sequence” is essential and sufficient for the full activation of Gpr54 receptor by kisspeptin in different vertebrate species. All forms of the kisspeptins have similar affinity to G protein-coupled receptor, GPR54 (12). It is proven that Kisspeptin/GPR54 signaling system plays a critical role in the regulation of the hypothalamic-pituitary-gonadal (HPG) axis. The GPR54 receptor is essential for normal pubertal development and sexual function (13, 14). It was shown that dysfunction or mutations in the gene encoding the GPR54 receptor, cause hypogonadotropic hypogonadism and pubertal delay in humans and rodents (13, 14). Also it was shown that the majority of GnRH neurons express the GPR54 receptor and central or peripheral administration of the kisspeptin potently stimulates the HPG axis. Moreover central or peripheral injection of the GPR54 receptor antagonist, peptide 234 significantly blocks the stimulatory effect of kisspeptin on HPG axis activity (15, 16); on the other hand, the arcuate nucleus (ARC) kisspeptin/neurokinin B (NKB)/dynorphin (KNDy) neurons play a role in the negative feedback effect of estrogen on the pulsatile release of gonadotropin-releasing hormone (GnRH)/luteinizing hormone (LH) (17, 18).

## 2. Objectives 

 Opiates are increasingly consumed as drugs of abuse or for the management of pain. Opiate consumption affects gonadal functions and induces hypogonadism, amenorrhea and infertility (8, 9). Also, previous studies have shown that kisspeptin/GPR54 signaling pathway exerts stimulatory effects on HPG axis activity and some suggest kisspeptin peptides as candidate drug for the management of infertility (13-16). Several lines of evidence suggest that endogenous opioid peptides and opiates (e. g. morphine) exert their inhibitory effects on the HPG axis via different interneurons (19, 20). However, in the above reports, solely the effects of opiate injection or kisspeptin injection alone on HPG axis were determined and we could not find any report on the interaction of morphine (the main opiate involved in the regulation of the HPG axis activity) and kisspeptin signaling pathway on the regulation of HPG axis. In the present study we aimed to investigate the effects of different doses of kisspeptin 10, P234 or co-administration of kisspeptin, morphine and P234 on the mean plasma testosterone concentration in male rats to understand if kisspeptin and morphine may interact with each other to control the reproductive axis, and if kisspeptine is a suitable candidate for pharmacological intervention of reproductive dysfunctions caused by opiates. 

## 3. Materials and Methods

### 3.1. Animals

In the present experimental study, male Wistar rats (n= 70) weighing 230-250 g (provided by the Center for Neuroscience Research of Shahid Beheshti University, Iran) were housed in cages under controlled temperature (22± 2 C°) and light (12h light/dark cycle, light on 0700h). Animals had free access to food and water all the time. All procedures for the maintenance and use of experimental animals were approved by the ethics committee of Neuroscience Research Center of Shahid Beheshti University of Medical Sciences (Tehran, Iran).

### 3.2. ICV Cannulation and Injections

Animal surgical procedures and handling were carried out as previously described (21). Animals were anesthetized by intraperitoneal (IP) injection of a mixture of ketamine and xylezine (ketamine 80 mg/kg BW+ xylezine 10 mg/ kg BW). For central injections, a 22-gauge stainless cannulae was implanted in the third cerebral ventricle according to the coordinates of Paxinos and Watson Atlas (AP = - 2.3, ML=0.0, DV = 6.5). The cannula was secured to the skull with three stainless steel screws and dental cement. Animals were kept in individual cages. After a one week recovery period, seventy rats in 14 groups (n = 5 in each group) received saline (3 µL, ICV)/ saline (200 µL, sc), kisspeptin (100 pmol, 1 or 3 nmoL/3 µL, ICV)/ saline (200 µL, sc), P234 (1 or 2.5 nmol/3 µL, ICV)/saline (200 µL, sc), kisspeptin (1 nmol/1.5 µL, ICV)+ P234 (1 or 2.5 nmol/1.5 µL, ICV)/saline (200 µL, sc), kisspeotin (1 nmol/3 µL, ICV)/ morphine (5 mg/kg, 200 µL, sc), kisspeptin (1 nmol/3 µL, ICV)/ naloxone (2 mg/kg, 200 µL, sc), P234 (1 nmol/3 µL, ICV)/morphine (5 mg/kg; 200 µl, sc), P234 (1 nmol/3 µL, ICV)/naloxone (2 mg/kg; 200 µL, sc), kisspeptin (1 nmol/1.5 µL, ICV)/+ P234 (1 nmol/1.5 L, ICV)/morphine (5 mg/kg; 200 µL, sc), kisspeptin (1 nmol/1.5 µL, ICV)/+ P234 (1 nmol/1.5 µL, ICV)/naloxone (2 mg/kg; 200 µL,sc). Kisspeptin, P234, morphine and naloxine doses were chosen based on previous studies, which had established their stimulatory or inhibitory effects on HPG axis (2-5, 15-16, 22-25). For ICV injection, kisspeptin 10 (Ana Spec Co, USA) and P234 (Phoenix Pharmaceutical Co, USA) were dissolved in DMSO and saline, respectively. They were injected by a 27-gauge stainless steel injector (protruded 0.5 mm beyond the cannula), which was connected to a Hamilton micro syringe by PE- 20 tubing 0at 09:00- 09:30. For subcutaneous injection, morphine sulphate (Temad Co, Iran) and naloxone hydrochloride (Toliddaru Co, Iran) were dissolved in distilled water and injected by an insulin syringe at 09:00- 09:30 am. In co-administrated groups, P234, morphine or naloxone were injected 15 minutes before kisspeptin 10 injections. 

### 3.3. Hormone Assays and Statistical Analysis

Blood samples were collected in a volume of 0.5 mL at 60 minutes following injections (22, 23). Heparin was added to the samples to prevent clotting. Blood samples were immediately centrifuged for 15 minutes at 3000 rpm and plasma was stored at –20°C until assayed for testosterone concentration. Plasma testosterone concentration was measured by using rat testosterone kit and the method of the enzyme-linked immunosorbent assay (ELISA) (Bitamed. Co, Iran). Sensitivity, intra- and inter-assay coefficients of variation of the kit were 0.06 ng/mL, 6.61% and 9.32%, respectively. Results are presented as mean ± SEM. Data were analyzed by one-way ANOVA test followed by post hoc Tukey’s test using the SPSS software (version 16). In all cases, significant difference was defined as P < 0.05.

## 4. Results

**Figure 1.**
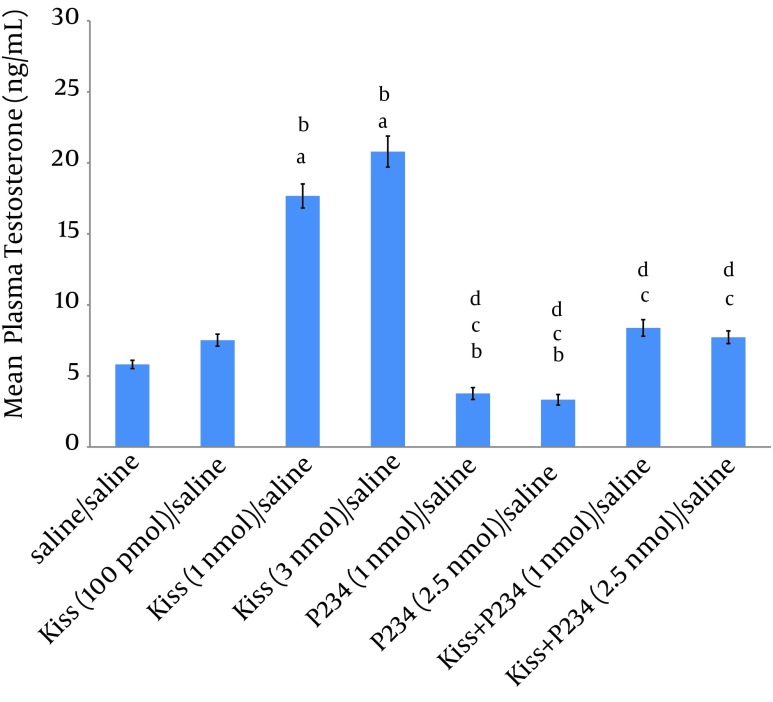
Effects of Kisspeptin, P234 or Co-Administration of Kisspeptin and P234 on Mean Plasma Testosterone Concentration in Male Wistar Rats Significant differences are indicated by letters; a: compared to saline/saline group; b: compared to kisspeptin (100 pmol)/ saline group; c: compared to kisspeptin (1 nmol)/saline group; d: compared to kisspeptin (3 nmol)/saline group (data presented as mean ± SEM, P < 0.05, n = 5 in each group).

The results showed that 100 pmol kisspeptin did not significantly increase the mean plasma testosterone concentration compared to saline/saline group (Figure 1). Injection of 1 nmol or 3 nmol kisspeptin/saline significantly increased the mean plasma testosterone concentration compared to saline/saline or kisspeptin (100 pmol)/saline group. Also, a significant difference was observed between the effect of kisspeptin (1 nmol)/saline and kisspeptin (3 nmol)/saline on the mean testosterone concentration (Figure 1). Injection of 1 or 2.5 nmol P234/saline decreased the mean plasma testosterone concentration compared to the saline/saline group but this decrease was not statistically significant (Figure 1). Also, no significant difference was observed between the effects of 1 nmol and 2.5 nmol of P234 on mean testosterone concentration (Figure 1). Co-injections of kisspeptin (1nmol) and P234 (1 or 2.5 nmol) significantly decreased the mean plasma testosterone concentration compared to kisspeptin (1 nmol)/saline group (Figure 1), but a significant difference was not observed between kisspeptin (1 nmol) + P234 (1 nmol)/ saline and kisspeptin (1 nmol) + P234 (2.5 nmol)/ saline group effects on mean testosterone concentration (Figure 1). 

**Figure 2.**
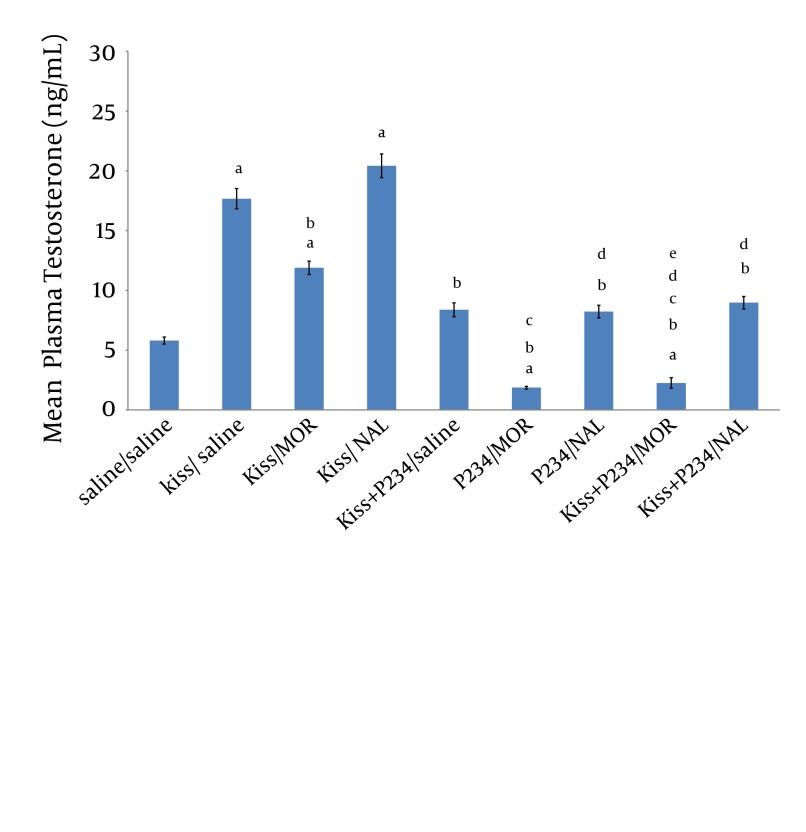
Effects of Co-Administration of kisspeptin (1 nmol), P234 (1 nmol), Morphine (5 mg/kg) or Naloxone (2 mg/kg) on Mean Plasma Testosterone Concentration in Male Wistar Rats Significant differences are indicated by letters; a: compared to saline/saline group, b: compared to kisspeptin (1 nmol)/saline group, c: compared to kisspeptin (1 nmol)/morphine (5 mg/kg) group, d: compared to kisspeptin (1 nmol)/naloxone (2 mg/kg) group, e: compared to kisspeptin (1 nmol) +P234 (1 nmol)/ saline group (data presents mean ± SEM, P < 0.05, n = 5 in each group).

The results revealed that mean plasma testosterone concentration was significantly decreased following co-administration of kisspeptin (1 nmol)/morphine (5 mg/kg) compared to the kisspeptin (1 nmol)/saline group (Figure 2). Mean testosterone concentration was increased after co-administration of kisspeptin (1 nmol)/naloxone (2 mg/kg) compared to the kisspeptin (1 nmol)/saline group but this increase was not statistically significant (Figure 2). Mean plasma testosterone concentration did not significantly increase after co-administration of kisspeptin (1 nmol)/ P234 (1 nmol) compared to saline/saline group (Figure 2). Testosterone concentration was significantly decreased after co- administration of kisspeptin (1 nmol)/P234 (1 nmol) compared to the kisspeptin (1 nmol)/saline group (Figure 2). Mean plasma testosterone concentration was significantly decreased following co-administration of P234 (1 nmol)/ morphine (5 mg/kg) compared to the saline/saline, kisspeptin (1 nmol)/saline group or kisspeptin (1 nmol)/morphine (5 mg/kg)/group (Figure 2). Testosterone concentration was significantly decreased following co-administration of P234 (1 nmol)/ naloxone (2 mg/kg) compared to the kisspeptin (1 nmol)/saline group or kisspeptin (1 nmol)/ naloxone (2 mg/kg) group (Figure 2). Mean plasma testosterone concentration was significantly decreased following co-administration of kisspeptin (1 nmol) + P234 (1 nmol)/ morphine (5 mg/kg) compared to the kisspeptin (1 nmol)/saline or kisspeptin (1 nmol)/ morphine (5 mg/kg) or kisspeptin (1 nmol) +P234 (1 nmol)/ saline group (Figure 2). Mean plasma testosterone concentration was significantly decreased following co-administration of kisspeptin (1 nmol) + P234 (1 nmol)/ naloxone (2 mg/kg) compared to the kisspeptin (1 nmol)/saline group or kisspeptin (1 nmol)/ naloxone (2 mg/kg) group (Figure 2). However, the testosterone concentration did not significantly decrease following co-administration of kisspeptin (1 nmol) + P234 (1 nmol)/ naloxone (2 mg/kg) compared to the P234 (1 nmol)/ naloxone (2 mg/kg) group (Figure 2). 

## 5. Discussion

The doses of kisspeptin used in the present study significantly increased the mean plasma testosterone concentration compared to saline in a dose-dependent manner. These results are in agreement with previous studies, which established that kisspeptin/GPR54 signaling system is a key component of reproduction and both central and peripheral administration of kisspeptins increases plasma LH, FSH and total testosterone directly at the hypothalamic but not the pituitary level (12-16). Also, our results proved that different doses of P234 significantly inhibited the stimulatory effects of kisspeptin on testosterone secretion. These data are in agreement with previous studies showing that more than 85 percent of GnRH neurons express GPR54 receptor and both intra ICV or arcuate nucleus (ARC) injections of P234 significantly decrease the mean plasma testosterone concentration and block the stimulatory effects of kisspeptins on the HPG axis (15, 16).

It has been demonstrated, in the previous studies, that the inhibitory effects of opioids on HPG axis is mediated mainly via hypothalamic GnRH rather than direct effects on the pituitary gonadotrophs (5, 7, 10, 11). However, the precise mechanism of the inhibitory effects of opioids on the reproductive axis is controversial and the majority of previous studies have showed that endogenous opioids or exogenous opiates (e. g. morphine) and their antagonists, influence the release of GnRH/LH and subsequently gonadal steroid hormone secretion via different hypothalamic interneurons. In fact, they reported that, especially down regulation of noradrenergic or up regulation of GABAergic neurons, play an important role in relaying the indirect effects of opioids and their antagonists on the hypothalamic GnRH-producing neurons (19, 20). Recent studies have shown that there is an interaction between kisspeptin and dynorphin (an endogen opioid peptide) in controlling HPG axis (17, 18) but there has not been any report on the interaction of morphine and kisspeptin/GPR54 signaling system on the HPG axis until now. In the present study, we investigated the effects of interaction between P234 and morphine or naloxone on the mean plasma testosterone concentration. The results showed that P234 significantly attenuated testosterone response to naloxone injection compared to kisspeptin/naloxone and co-administration of morphine/P234 significantly decreased testosterone secretion compared to kisspeptin/morphine or kisspeptin/P234. As the effects of interaction of morphine and P234 were investigated on reproductive axis for the first time, there were no previous studies to compare the results. However morphine may affect the amplitude of kisspeptin/GPR54 signaling pathway activity. Recently, it has been established that some important factors involved in the control of sexual function including steroid hormones, fasting or ghrelin exert inhibitory effects on HPG axis via down regulation of the kisspeptin/GPR54 signaling system. Ghrelin injection results in a significant decrease in Kiss1 gene mRNA level in the brain (24-27). Also it has been revealed that leptin or even photoperiod (in seasonal animals) exert their stimulatory effects on the reproductive axis via up-regulation of kisspeptin gene expression (28). Because most recent studies reported that the kisspeptin signaling system is involved in reproductive pathways, and in this study we demonstrated that injection of morphine/P234 abolishes the stimulatory effects of kisspeptin on testosterone secretion, one can speculate that morphine may interact with the kisspeptin system to control the HPG axis. In our future studies we will try to examine the interaction of morphine, naloxone or their simultaneous injection on Kiss1 or kiss1r (GPR54 receptor) gene expression level or other inhibitory or stimulatory factors involved in reproduction like ghrelin and leptin, to understand if opioids may directly or indirectly interact with the kisspeptin system in controlling the HPG axis activity.

 Results of the present study showed that central injection of kisspeptin 10 significantly increased the mean plasma testosterone concentration compared to saline and co-administration of morphine/P234 or naloxone/p234 significantly decreased testosterone secretion; thus morphine and kisspeptin systems may interact with each other to control the hypothalamic-pituitary-gonadal axis in rats.
